# EEG Biomarkers for Stress Detection: A Comparative Analysis of Hjorth Parameters, Spectral and Fractal-Dimension Measures

**DOI:** 10.3390/s26216978

**Published:** 2026-11-04

**Authors:** Michael Leitner, Kyriaki Kostoglou, Selina C. Wriessnegger

**Affiliations:** Institute of Neural Engineering, Graz University of Technology, Stremayrgasse 16/IV, 8010 Graz, Austria; michael.leitner@tugraz.at (M.L.); kkostoglou@tugraz.at (K.K.)

**Keywords:** EEG, discriminative power, fractal dimensions, stress classification, machine learning

## Abstract

EEG-based stress detection is increasingly relevant for intelligent sensing and human–computer interaction, particularly for systems that aim to adapt to users’ changing cognitive and affective states. While conventional EEG-based stress detection approaches mainly rely on spectral band-power features, fractal-dimension measures and Hjorth parameters may provide additional information about underlying brain dynamics. However, their relative contribution, spatial distribution, and frequency-band dependence remain insufficiently understood. In this study, pre-recorded EEG data from a stress-inducing experiment were analyzed using statistical feature pre-selection and multiple machine-learning classifiers. The contribution of different EEG features was evaluated across participants, while scalp topographical maps were used to characterize the spatial distribution of informative features. The results identify Hjorth activity, frontal β asymmetry, and the γ/θ ratio as among the most frequently selected features in the ANOVA-based feature-selection analysis. Combined feature-space configurations including Hjorth parameters and fractal-dimension measures achieved higher classification performance than the conventional-feature reference configuration. These findings provide further insight into the spatial and frequency-specific characteristics of EEG features associated with the investigated stress-inducing cognitive task and demonstrate the value of combining conventional spectral features with Hjorth parameters and fractal-dimension measures for physiological-state sensing. The proposed approach may support the development of computationally efficient EEG-based monitoring of stress-related cognitive states for intelligent and adaptive human–computer interaction, including neuroadaptive and context-aware systems.

## 1. Introduction

In recent years, the rapidly changing world has introduced an increasing number of factors that promote stress. According to the stress theory proposed by Lazarus & Folkman [1], stress arises from an imbalance between a person’s actual abilities and their perceived abilities in relation to a task or situation. When individuals underestimate their own abilities, stress is experienced, affecting both brain activity and the sympathetic nervous system. While common causes include threats or external pressures, stress is also encountered in many everyday situations. Individual stressors, such as work overload [2,3] or techno-stress [4,5] can accumulate and contribute to mental and cardiovascular disorders [6,7,8].

Given the widespread impact of stress and its adverse effects on health, reliable methods for assessing stress levels are essential. In many settings, stress is still evaluated through self-reports or verbal feedback provided to a therapist or experimenter. However, such subjective and temporally sparse assessments do not provide a stable basis for monitoring continuous stress dynamics. Physiological parameters, in contrast, are more consistent and precise and can be tracked in real time without interrupting the subject. Among these, neurophysiological recordings such as the electroencephalogram (EEG) capture stress-related brain activity directly and offer a more objective assessment. In experimental studies, stress is commonly induced through paradigms such as mental arithmetic or the Montreal Imaging Stress Task [9,10], incorporating time pressure and negative feedback. Additional exposure scenarios include specific stressors such as arachnophobia [11], glossophobia [12], or claustrophobia [13]. Based on such paradigms, machine learning approaches have increasingly been applied to EEG recordings to automatically detect stress states [14]. A central challenge in EEG-based stress detection remains the identification of reliable features that represent robust stress markers for classification.

### 1.1. EEG Features for Stress Classification

The EEG features commonly used in mental stress detection primarily include band power (BP) features in the standard frequency bands, with the θ [15] and β [16] bands identified as highly relevant stress markers. Furthermore, suppression of α activity has been widely reported during stress-related conditions [17,18,19]. These studies also report stress-associated changes in the β band, further supporting its relevance for stress detection. In general, the most commonly extracted EEG features include relative band power (rBP) [13,17], absolute band power (BP) [12,19,20], and BP ratios [15,16]. These BP-based measures (including relative or absolute BP, asymmetry indices, and BP ratios) are well established and are often complemented by statistical, time-domain, and complexity-related descriptors of the EEG signal. These additional descriptors characterize temporal variability, frequency characteristics, and signal complexity beyond conventional band-power measures. Temporal complexity features such as fractal dimension (FD) measures have also been shown to capture stress-related dynamics and to contribute to high classification performance [21]. FD measures quantify changes in signal complexity associated with different mental states and therefore support their use as stress markers in classification tasks.

Among commonly used time-domain descriptors, the Hjorth parameters [22] are typically reported as a triplet consisting of Hjorth Activity (HAC), Hjorth Mobility (HMB), and Hjorth Complexity (HCP). HAC reflects the mean signal power or energy and has been shown to correlate with band-specific BP features [23,24]. In particular, rBP and HAC are closely related, as HAC captures overall amplitude fluctuations in the signal [23,25,26]. HMB approximates the mean frequency characteristics or average rate of change [24], while HCP quantifies the deviation from a pure sinusoidal waveform and is therefore interpreted as a measure of bandwidth and signal irregularity [25].

Additional FD based features used in EEG analysis include the Higuchi Fractal Dimension (HFD) [27]. HFD captures multi-scale temporal complexity or roughness and has been applied successfully in cognitively demanding tasks [28]. The Katz Fractal Dimension (KFD) [29] has likewise been successfully used as an EEG feature and characterizes waveform tortuosity, providing an alternative description of temporal complexity [30]. A more recent feature is the Petrosian Fractal Dimension (PFD) [31], originally proposed for the analysis of epileptic EEG signals. By quantifying the number of sign changes in a signal, PFD captures rapid fluctuations or irregular transitions. This approach is able to capture nonlinear and non-stationary aspects of EEG signal complexity [32].

All FD measures employ different formulations to characterize the complexity of a time series and have demonstrated strong performance in various EEG-based classification tasks. Depending on their methodological definition, these features may capture changes related to signal amplitude, spectral power, or frequency content. However, most existing studies estimate FD measures from the broadband EEG signal rather than from band-pass-filtered signals. This makes it difficult to determine whether the discriminative information originates from specific neural band power changes (e.g., θ, α, or β) or from broadband signal properties. Previous studies have not systematically examined how frequency-band contributions and scalp topography jointly affect the importance of FD measures in EEG-based classification. Furthermore, FD measures are sensitive to high-frequency signal components, making them particularly susceptible to contamination by electromyographic (EMG) activity and other high-frequency artifacts.

### 1.2. Progress on Stress Classification Methods

A recent study [33] employed a conservative multi-modal approach, combining EEG features with commonly used electrocardiogram (ECG) features. Their results indicate improvements when integrating multiple physiological domains. However, their findings also suggest that the use of traditional features alone is not sufficient, particularly as they observe non-consistent performance measures. The reported results strongly depend on the applied classification method, which contrasts with findings reported in several recent studies. Several studies have reported average accuracies ranging from 76% to 95% consistently across participants and classifiers [13,16,23,24,34,35,36,37]. Several of these papers included FD measures and Hjorth parameters in their feature extraction pipelines and partially reported relevant improvements. In [34], it was observed that HFD outperforms established features such as wavelet-based and BP features and performs even more robustly when combined with these established features, achieving around 85% accuracy. A second study indicated the strong performance capabilities of HFD and KFD [21]. However, PFD and HFD were reported to be more sensitive to noise, while KFD was found to exhibit slightly greater robustness [34]. Overall, these findings suggest that combining FD measures with band power features, rather than relying on a single feature type, may better capture significant stress-related effects across relevant brain regions. Specifically, classification approaches which included Hjorth parameters, as reported in [23,24], achieved accuracies of 76% and 80%, respectively. Their methods also indicated that feature pre-selection led to substantial improvements, yielding consistent accuracy gains across all applied machine learning algorithms. In comparison to FD measures, ref. [37] used PFD to classify difficulty stages in a Color Word and Memory Test and reported significant changes in PFD between experimental stages. As their study was limited by the number of sensors, no clear topographical conclusions could be drawn; however, the effectiveness of PFD was demonstrated. The study of [34] used wavelet features, KFD, and HFD and achieved accuracies of up to 83%. In their comparison, no specific FD algorithm emerged as superior to band-specific wavelet features. The study in [36] reported fatigue classification accuracies of approximately 95%, representing an improvement of 7–8% over previously reported approaches.

The current state of the art suggests achievable accuracies of at least 80% (binary classification) and above, depending on the hardware, study design, classifier and, most importantly, the selected feature set. Several of the previously mentioned studies report that statistical feature pre-selection improves classification performance in machine learning algorithms. In a preliminary study, ref. [38] reported promising stress classification results by extracting a large number of features and training commonly used classifiers to detect mental workload and stress. Their results demonstrated that elevated stress can be detected with high accuracy compared to resting periods. Another finding was that training on more than 100 features may negatively influence classification accuracy. In [38], an ANOVA-based feature ranking was also applied to improve classification performance. A subsequent study by [35] indicated significant differences between sexes and further demonstrated that machine learning algorithms are capable of achieving high individual-level accuracy across multiple stress levels. As their method focused on personalization rather than generalization, this approach may represent one step toward achieving greater stability in mental state detection. An open question left by both [35,38] however is the feature discrimination strength especially in this individualization pipeline. Several of the aforementioned studies report findings comparable to those presented in [39], while maintaining relatively low computational cost. Additional evidence is provided by [40], which supports the effectiveness of combining FD measures with band power (BP) features. However, that work does not evaluate the relevance of individual features or their associated topographical relationships.

A broad spectrum of classifiers has been investigated in the field of mental stress detection. The most common methods include deep learning algorithms which utilize convolutional neural network architectures as well as machine learning algorithms [41,42]. Both studies compared various algorithms from both families and found classification performance (accuracy) on a comparable level from 60% up to 80% in binary classification problems. As summarized in [43,44,45,46,47], shrinkage Linear Discriminant Analysis (sLDA), Support Vector Machines (SVM), k-nearest neighbors, Random Forests (RF), Gaussian Naive Bayes (NB), and neural networks implemented as Multi-Layer Perceptrons (MLP) are among the most frequently reported classifiers performing on a consistent high accuracy level. The recent work of [33] highlighted substantial performance differences between classifier families; however, most of the previously mentioned studies demonstrated consistently strong performance (i.e., above 80% accuracy) across classifiers. In many cases, decision trees have largely been superseded by RF and boosting algorithms such as AdaBoost or gradient boosting. Across studies, classifiers also tend to exhibit relatively consistent performance across different feature sets. Nevertheless, it remains unclear whether discriminative power of features varies across electrode positions. This question is particularly relevant because many studies perform classification using a limited number of electrodes, often eight or fewer [9,12,15,48,49,50]. Consequently, potential spatial differences in feature relevance may remain insufficiently explored.

Table 1 summarizes representative EEG-based stress-classification approaches and highlights the considerable diversity in experimental paradigms, EEG configurations, feature representations, and classification methods.

Most previous work has primarily focused on classification performance using spectral features, asymmetry measures, connectivity estimates, or selected complexity-related descriptors. FD measures have also been investigated for stress classification, although they have commonly been evaluated either as broadband descriptors or as part of comparatively restricted feature sets. In contrast, the present study focuses on a systematic comparison of spectral, statistical, and FD measures within the same analysis framework. In particular, we investigate their frequency-specific and spatial selection patterns across the scalp, compare broadband and band-specific FD measures, and evaluate the resulting feature sets across multiple classifier families. This allows the study to address not only classification performance but also how different EEG feature families are distributed across frequency bands and electrode locations.

### 1.3. Research Gap

Most studies prioritize classification accuracy over neurophysiological interpretability [53,54]. Consequently, several important questions remain unresolved, including the spatial relevance of established EEG features and FD measures, the frequency-specific contributions of spectral and complexity-based features, and the extent to which FD measures provide additional information beyond conventional spectral biomarkers. Although FD measures are frequently combined with other EEG features, particularly in studies using reduced electrode setups [14,53], their individual importance has rarely been assessed systematically. To the best of our knowledge, few studies have directly assessed the discriminative power of FD measures with established physiologically interpretable EEG biomarkers, including band-power (BP), BP ratios, and asymmetry measures, which are consistently associated with stress or cognitive demand [14,53,55]. Furthermore, the robustness of FD measures following rigorous artifact removal and feature selection remains unclear because of their sensitivity to preprocessing and high-frequency signal components. Finally, the extent to which Hjorth parameters and FD measures improve classification performance beyond conventional spectral features has been poorly investigated.

### 1.4. Goal and Hypothesis

As a first objective, we focus on Hjorth parameters and FD measures and their selection frequency in stress classification and assess their contribution to classification performance. To what extent does the inclusion of FD measures improve classification performance? A key unresolved question concerns the spatial distribution of discriminative power when Hjorth parameters and FD measures are incorporated alongside BP-based features. As second objective, we aim to systematically explore the band-specific feature selection frequency of rBP, BP ratios and asymmetries as well as FD measures in their spatial distribution. As third objective, we investigate how the number of extracted features influences classification performance and whether a limit exists beyond which adding additional features no longer improves accuracy. To address this, we analyze the relationship between the number of features retained after pre-selection and the resulting classification performance across multiple classifier algorithms.

FD measures are typically computed over the full EEG frequency spectrum. Applying FD measures to individual EEG frequency bands while simultaneously examining their spatial selection frequency across electrodes has not been systematically investigated in EEG-based stress classification. Although band-pass filtering changes the interpretation of FD measures, it allows the complexity of individual oscillatory components to be examined, helping to determine whether the discriminative information arises from specific neural rhythms or from broadband EEG dynamics. Accordingly, the resulting measures should be interpreted as indices of within-band temporal complexity rather than global EEG fractality.

We hypothesize that including FD measures and Hjorth parameters alongside conventional spectral features improves classification performance and changes the composition of the most frequently selected feature subsets. However, we expect physiologically interpretable measures, such as spectral power, power ratios, and asymmetry metrics, to emerge as markers with strong separability between the investigated stress phases.. Furthermore, we hypothesize that stress-related features will exhibit stable selection patterns over individual participant iterations, and resilience against common EEG artifacts.

Understanding why classifiers perform well is increasingly important for identifying physiologically meaningful biomarkers and improving trustworthiness of EEG-based stress detection systems. This study is designed to identify EEG features that may support individualized classification within the present stress-inducing experimental paradigm and provide a basis for future real-world investigations. This requires low variance, low sensibility to artifacts and spatial consistency.

## 2. Methods

### 2.1. Experimental Paradigm

The analysis was performed using a pre-recorded dataset from our previous study [56]. The dataset comprised recordings from 30 participants (16 males, 14 females), with each dataset corresponding to one distinct participant. The study was approved by the ethics committee of Graz University of Technology (GZ/2024).

**Figure 1:**
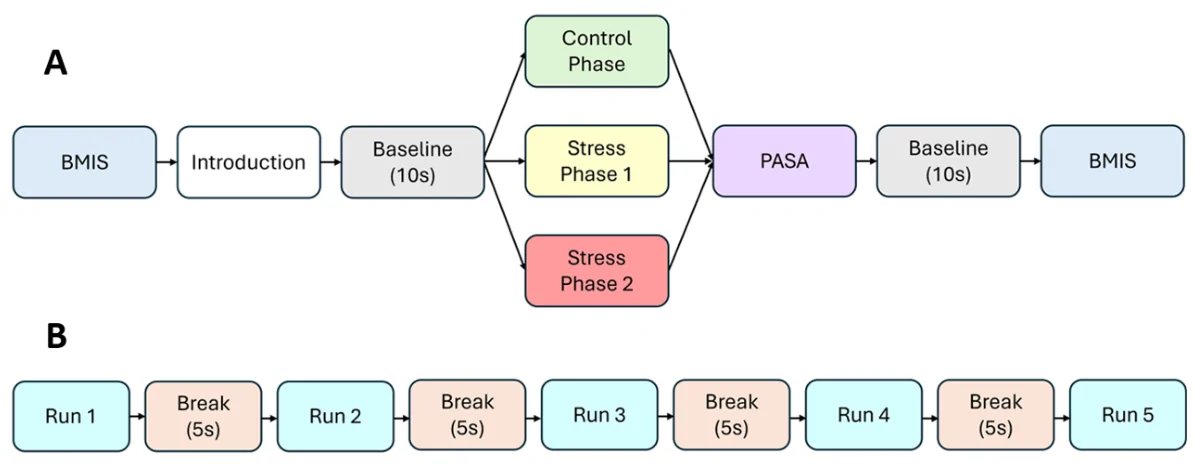
Experimental procedure from [56]: (**A**) The overall experimental procedure during the measurement. In (**B**), a single trial from the mental arithmetic paradigm is displayed. Taken with permission from [56] under the Creative Commons CC-BY license.

The underlying paradigm consisted of a mental arithmetic task, as illustrated in Figure 1, which participants performed under time pressure. Each trial consisted of a 15-s baseline recording (relaxing with open eyes), a calculation sequence, and a second baseline recording. The calculation sequence comprised three conditions (control, stress phase 1, and stress phase 2). In the control phase, arithmetic tasks were presented without time pressure or additional noise. In both stress phases, environmental background noise (office, café) was added, and responses had to be provided within a time-limited window. The maximum response duration was set to 90% of the participant’s average response time in the control phase. Stress phase 2 differed from stress phase 1 by an increased number of arithmetic operations and the use of larger digits. Each block (control, stress phase 1, and stress phase 2) was repeated five times, resulting in a total of 225 trials per participant (5 runs × 15 trials × 3 conditions).

Before and after the experiment, participants completed the brief mood introspection scale (BMIS) [57], while subjective stress was assessed using the primary appraisal–secondary appraisal (PASA) questionnaire [58]. These measures were analyzed in the original study [56]. The BMIS showed a significant decrease in overall mood following the experiment, while PASA stress scores differed significantly between the control condition and both stress phases, as well as between Stress Phase 1 and Stress Phase 2. These subjective measures therefore support successful stress induction across the experimental manipulation.

### 2.2. Data

The dataset from [56] comprised EEG recordings from 30 unique participants, aged 19 to 32 years (mean: 25.08 ± 3.4 years). EEG signals were recorded using the LiveAmp system (BrainProducts GmbH, Gilching, Germany) at 500 Hz sampling rate. Electrode placement followed the international 10–20 system, and electrode impedances were maintained below 20 kΩ throughout the recording. Due to consistently high noise levels observed in electrodes TP9, TP10, FT9, and FT10 across all datasets, these channels were excluded from further analysis. Consequently, signals from the remaining 28 EEG channels were processed in the subsequent analysis (Fp1, Fz, F3, F7, FC5, FC1, C3, T7, CP5, CP1, Pz, P3, P7, O1, Oz, O2, P4, P8, CP6, CP2, Cz, C4, T8, FC6, FC2, F4, F8, Fp2). All physiological signals, together with paradigm event markers, were acquired and synchronized using the Lab Streaming Layer (LSL).

### 2.3. Preprocessing

All analyses were performed using Python 3.10 on a MacBook Air M4 (2025) with the following libraries: MNE-Python = 1.6.0, SciPy = 1.11.0, NumPy = 1.26.0, scikit-learn = 1.4.0, PyTorch = 2.5.1, mne-features = 0.3.1, pandas = 2.2.0, statsmodels = 0.14.0, and pyxdf = 1.16.5.

**Figure 2:**
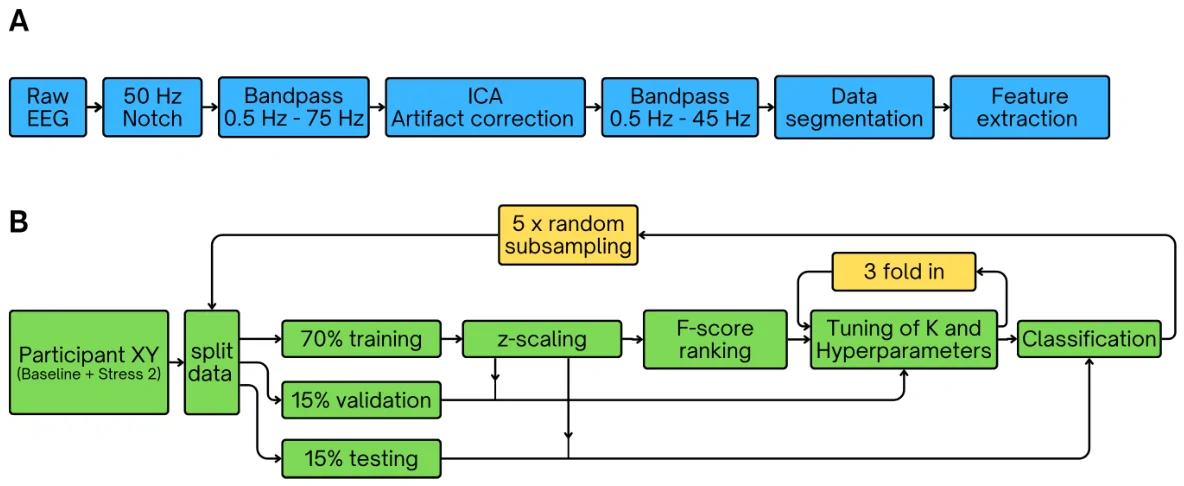
(**A**) Preprocessing pipeline including filtering, artifact rejection, and data segmentation. (**B**) Subject-dependent classification pipeline including the within-participant train–validation–test split, z-score normalization, ANOVA-based feature ranking and K-Best selection, 3-fold inner hyperparameter optimization, and evaluation on the held-out test set. The complete procedure was repeated for five independent random partitions per participant.

The electrode setup followed the international 10–20 system, of which 28 EEG channels were included in the analysis. An overview of the preprocessing steps is provided in Figure 2. All signals were filtered using zero-phase fourth-order Butterworth filters and a 50 Hz notch filter. EEG data were initially bandpass-filtered between 0.5 Hz and 75 Hz. Independent Component Analysis (ICA; infomax, 18 components) was applied, and components associated with muscle and eye-movement artifacts were manually rejected. Eye-related components were identified based on their topography and characteristic properties (frontal distribution/asymmetry and characteristic time series). Electromyography (EMG) artifacts were additionally analyzed based on their PSD slope (frontal and temporal activity islands, increasing slope, and characteristic time course) to determine rejection. After artifact correction, a final bandpass filter (zero-phase fourth-order Butterworth) from 0.5 Hz to 45 Hz was applied. Data were re-referenced to the common average. EEG data were segmented from 0.9 s to 3 s relative to trial onset for feature extraction. Epochs exceeding a maximum peak-to-peak amplitude of 130 μV or falling below 1 μV were discarded (average rejection rate: 10%).

### 2.4. Feature Extraction and Selection

The library MNE-Python [59] was used for data handling, visualization, and feature extraction. Features were calculated using all 28 electrodes. For frontal asymmetry values (α- and β-bands), the following electrode pairs were used: F7/F8, FC5/FC6, F3/F4, Fp1/Fp2, and FC1/FC2. In the frequency domain, relative band power (rBP) was computed from the power spectral density (PSD). rBP was estimated using Welch’s method (segments of 500 samples, Hamming window, FFT length 512 samples, 50% overlap). The analysis was performed for the δ (0.5–3.5 Hz), θ (4–7.5 Hz), α (8–12.5 Hz), β (13–29.5 Hz), and γ (30–45 Hz) bands. The use of rBP reduces inter-individual variation and mitigates biases associated with decreasing spectral power at higher frequencies [60,61]. Moreover, rBP provides higher sensitivity than absolute power. We also used the already existing bandpower values to compute the ratios of α/β BP (RAB), θ/β BP (RTB), and γ/θ BP (RGT). We additionally computed Hjorth parameters (HAC, HMB, HCP) [22] and FD measures using the algorithms of Katz (KFD) [29], Petrosian (PFD) [31], and Higuchi (HFD) [27]. FD measures were calculated for both the entire spectrum (0.5–45 Hz) and separately for each sub-band (δ, θ, α, β, γ). FD measures were computed on both broadband and band-pass-filtered EEG.

All features were calculated per epoch and per participant, the relevant formulas are summarized in Table 2. To investigate the potential influence of muscle artifacts commonly present in ring electrodes [62,63], an additional feature set was created for both the sub-band and full-band feature sets by excluding the ring electrodes (T7/T8, O1/O2, Oz, P7/P8), resulting in 21 remaining electrode positions. This resulted in a tripled classifier evaluation, combining two electrode configurations (including or excluding ring electrodes) with varying FD feature types (broadband and band-specific). Features were computed independently for each electrode, and each electrode–feature pair was treated as a separate input feature for the classifier. The following feature configurations were examined: no ring electrodes including band-specific features (21 electrodes per map, 38 maps, additional 10 asymmetry features = 808 features available); all electrodes including band-specific features (28 electrodes per map, 38 maps, additional 10 asymmetry features = 1074 features available); and all electrodes including band-specific features, but excluding the γ band (28 electrodes per map, 30 maps, additional 10 asymmetry features = 850 features available). These configurations were designed to investigate how electrode selection and frequency-band feature inclusion influence feature relevance and classification performance [26]. The exclusion of peripheral/ring electrodes and, separately, the γ band provided sensitivity checks for potential influences of peripheral and high-frequency artifacts. These sensitivity analyses probe dependence on peripheral electrode locations and high-frequency information, respectively, but do not permit attribution of the remaining signal to cortical or myogenic sources.

Z-score normalization was applied separately for each participant. The mean and standard deviation were estimated from the training data and subsequently used to normalize the validation and test sets. Feature selection was performed by applying ANOVA on the training split, ranking features according to their *F*-values, and selecting the top-*k* features. Specifically, models were evaluated using varying numbers of top-ranked features, with k∈{10,20,30,40,50,75,100,125,150,175,200,250}. In addition, a configuration not including Hjorth parameters or FD measures (k=236) was added. These datasets were provided to each classifier; further processing is described in the following section.

### 2.5. Classifiers

Classification was performed independently for each participant. The two classes were drawn from the Stress Phase 1 and Stress Phase 2 conditions to maintain class balance and to provide a clear contrast between Stress Phase 1 and the strongest stress condition. The binary contrast was chosen because the primary objective of the present study was the comparison of EEG feature representations rather than the discrimination between multiple stress levels. For each participant, the data were randomly divided at the trial level into training (70%), validation (15%), and test (15%) sets while preserving class balance. Z-score normalization was performed separately for each participant. The mean and standard deviation were estimated from the training data and subsequently applied to the validation and test sets (z-score normalization). Feature selection was performed on the training data using ANOVA F-scores. Features were ranked according to their F-values, and the top-*k* features were retained, with k∈{10,20,30,40,50,75,100,125,150,175,200,250}. A configuration containing only the 236 conventional features, without Hjorth parameters or FD measures, was additionally evaluated as a reference. The conventional 236-feature configuration served as a performance reference and was not intended as a dimensionality-matched ablation of individual feature families. Hyperparameter optimization according to Table 3, including optimization of *k*, was performed using a 3-fold inner split of the training data. The optimal configuration was selected based on the median validation accuracy across the inner folds. The selected hyperparameters and feature-set size were subsequently used to train the final model on the merged training and validation sets, and model performance was evaluated on the corresponding held-out test set. The complete train–validation–test procedure was repeated five times per participant using independent random partitions. Thus, the reported classification represents a subject-dependent, within-participant setting rather than a subject-independent or cross-participant evaluation.

The employed algorithms have previously been validated in [43] and are widely adopted in the literature. We compared shrinkage Linear Discriminant Analysis (sLDA), k-nearest neighbors (KNN), Support Vector Machines (SVM), Random Forests (RF), Gaussian Naive Bayes (NB), and a Multi-Layer Perceptron (MLP). Decision trees were not included because their classification accuracy was consistently approximately 10% lower than that of the other algorithms. All conventional machine-learning classifiers were implemented in Python 3.10 using the scikit-learn library.

The MLP was implemented using the PyTorch library and consisted of an input layer (input size × 64, ReLU, batch normalization, dropout 0.2), a hidden layer (64 × 32, ReLU, batch normalization, dropout 0.1), and an output layer (32 × 1). Classifiers were trained to maximize accuracy. For the MLP, validation loss was used for early stopping. We additionally applied a learning-rate scheduler (initial learning rate 1 × 10^−4^), the AdamW optimizer (betas 0.9 & 0.999, eps 1 × 10^−8^, weight decay 0.01), and BCE-with-logits loss. During validation, training was performed for a maximum of 100 epochs, whereas final training was performed for up to 200 epochs with a batch size of 32.

### 2.6. Sensitivity Analysis

To further assess the contribution of Hjorth parameters and FD measures to classification performance, a permutation-based sensitivity analysis was performed using all 1074 available features. The analysis was conducted independently for each participant using Stress Phase 1 and Stress Phase 2 as the two target classes. For each participant, a fixed stratified subset comprising 20% of the data was used for hyperparameter optimization by 3-fold cross-validation. Hyperparameters were selected according to the mean Matthews correlation coefficient (MCC) across folds, while feature scaling was fitted exclusively on the respective training folds. The resulting classifier-specific hyperparameters were retained for the subsequent sensitivity-analysis iterations.

For each participant, 100 repeated train–test iterations were performed. The fixed tuning subset was retained within the training data, while additional training trials and a new test set were randomly sampled from the remaining data to obtain an overall 80/20 training–test split. The same split was used for all classifiers within each iteration, and scaling parameters were estimated exclusively from the training data. Each fitted model was evaluated under three test conditions: unchanged test data containing all features, permutation of the complete FD-measure feature family, and permutation of the complete Hjorth-parameter feature family. For each feature family, a single shared row permutation was applied across all corresponding feature columns, thereby preserving the internal relationships within the respective family while disrupting its association with the class labels. Training data, spectral features, and test labels remained unchanged.

Sensitivity was quantified using the F1-score, with a larger decrease after permutation indicating stronger classifier reliance on the corresponding feature family. For statistical evaluation, F1-scores were first averaged across the 100 iterations for each participant. Pairwise differences between the three conditions were then assessed separately for each classifier using two-sided paired Wilcoxon signed-rank tests. Holm correction was applied across the 18 resulting comparisons (six classifiers × three pairwise condition comparisons).

### 2.7. Feature-Selection Frequency

Feature-wise heatmaps were generated to display the 50 highest-ranked features (type × position). To assess the stability of ANOVA-based feature selection across participants and repeated random splits, we counted how frequently each electrode–feature combination occurred among the 50 highest-ranked features. These selection frequencies were visualized as scalp maps. Accordingly, the resulting maps represent the stability and frequency of univariate ANOVA-based feature selection rather than classifier-specific or model-derived feature importance. High selection frequency therefore indicates that a feature repeatedly showed strong class-related differences according to its ANOVA F-score, but does not imply a unique or independent contribution to classification performance.

## 3. Results

**Figure 3:**
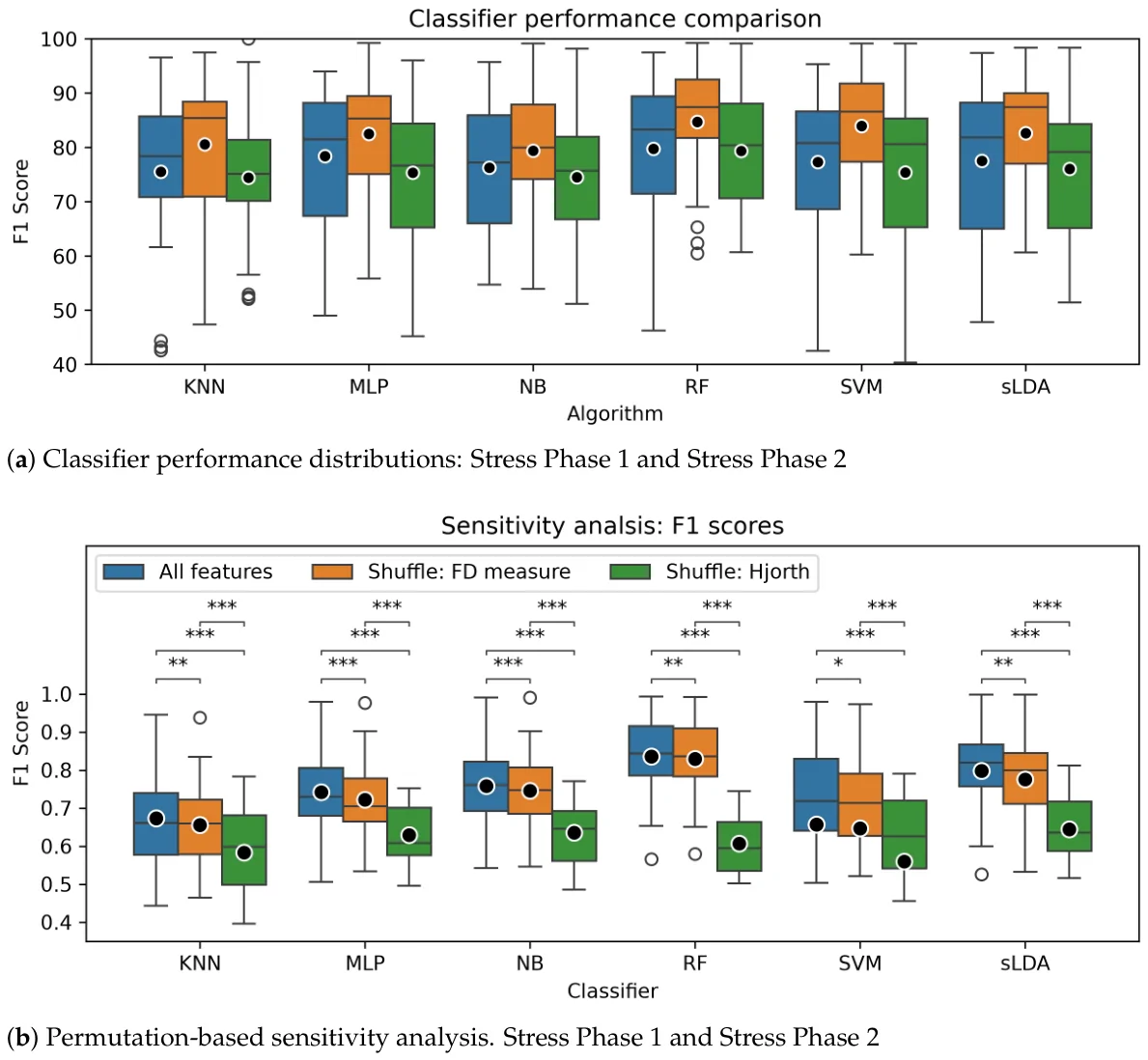
Classifier performance and permutation-based sensitivity analysis. (**a**) F1-score distributions across classifiers and feature configurations. Blue: no ring electrodes, band-specific features; yellow: all electrodes, band-specific features; green: all electrodes, band-specific features excluding the γ band. Black dots indicate mean F1-scores. (**b**) F1-score distributions across classifiers for all features (blue), shuffled FD measures (orange), and shuffled Hjorth parameters (green). Black circles indicate mean F1-scores. Brackets indicate significant pairwise differences (* p<0.05, ** p<0.01, *** p<0.001).

Figure 3a illustrates the performance distribution for all three feature configurations across all classifiers evaluated during the training iterations classifying stress phase 1 versus stress phase 2. Based on the average F1-score, the overall highest median F1 scores were obtained when using all electrodes and band-specific features. Within this split feature configuration, the highest average performance was achieved with RF (84.67% ± 10.53), closely followed by SVM (83.94% ± 10.23), sLDA (82.63% ± 11.31), and MLP (82.48% ± 10.55). The lowest F1-score within this configuration was obtained using NB (79.36% ± 11.32). This feature configuration is therefore identified as the overall best-performing configuration. Across all configurations, the lowest average F1-score was achieved by NB (74.39% ± 12.45). Complete results are provided in Table 4, where the two configurations with the highest F1-scores are highlighted. The reported F1-scores did not receive significant changes and are therefore not annotated in Figure 3a.

Figure 3b displays the F1-score distributions for the permutation-based sensitivity analysis. Across all classifiers, shuffling the Hjorth feature family produced a larger reduction in F1-score than shuffling the FD measures. With all features available, RF achieved the highest mean F1-score (0.84), followed by sLDA (0.80) and NB (0.76). Shuffling the FD measures resulted in comparatively small reductions across classifiers, with mean F1-scores remaining at 0.83 for RF and 0.78 for sLDA. In contrast, shuffling the Hjorth parameters reduced the mean F1-score to 0.61 for RF and 0.65 for sLDA, with the largest absolute reduction observed for RF. Similar, although smaller, decreases after Hjorth permutation were observed for the remaining classifiers.

**Figure 4:**
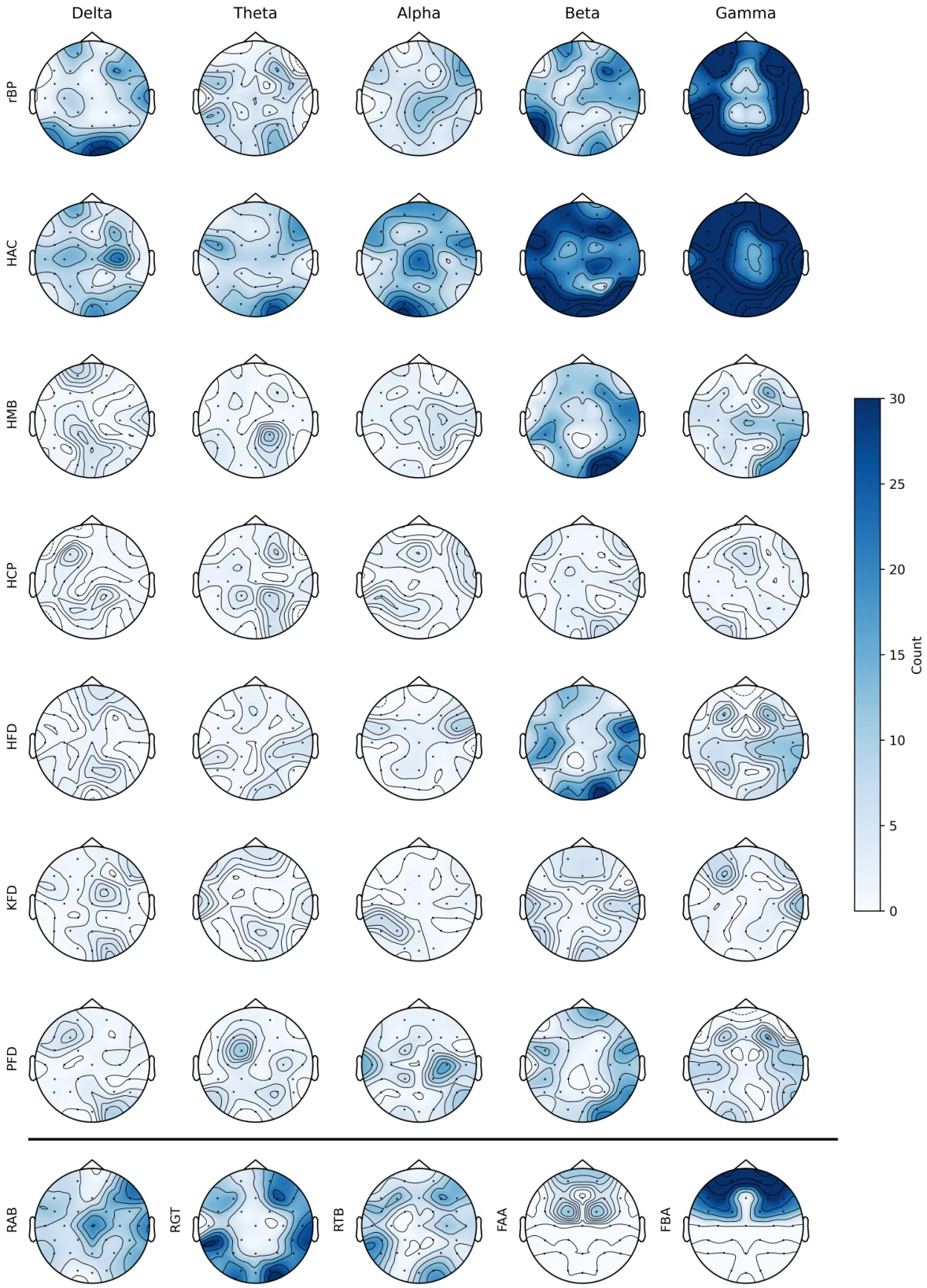
Feature-selection frequency maps for all electrodes and the band-specific feature evaluation of stress phase 1 versus stress phase 2. Counts indicate how often each electrode–feature combination occurred among the 50 highest-ranked ANOVA features across repeated random splits and participants. Counts are capped at 30 for visualization. The bottom row displays BP ratios and asymmetry measures.

Figure 4 displays feature occurrence maps in terms of selection counts across the full scalp. Each map represents one feature (rows) for a specific EEG band (columns). The bottom row contains the BP ratios and frontal asymmetry measures. All maps are based on the 50 highest-ranked features per repeated random split, resulting in 5 repetitions × 30 participants × 50 selected features = 7500 total selection counts. The largest evaluated feature configuration contained 1074 candidate features. Thus, across the complete evaluation, the number of selection positions corresponds to approximately seven potential occurrences per candidate feature on average. The analysis presented here refers to the configuration using all electrodes and band-specific FD measures. The overall most frequently selected features were rBP and HAC in the γ band, followed by β asymmetry. Approximately equally often selected were HAC in the α band, HMB and HFD in the β band, and RGT. rBP was regularly selected with a pronounced focus on frontal, temporal, and occipital electrodes. In the β band, a frontal asymmetry with right-hemispheric dominance was observed, whereas α showed central and parietal emphasis. θ did not exhibit a clear spatial preference, and δ, despite a right-dominant frontal focus, demonstrated strong occipital involvement. δ, θ, and α did not display consistent or prominent patterns in selection frequency across HMB, HCP, HFD, and KFD, nor strong similarities to rBP. In comparison, HAC is congruent with the selection frequency of the γ band and further amplified this effect. β-band selection frequency was primarily frontal and occipital, with increased counts in centro-parietal regions. In the α band, the primary focus was on frontal, central, and occipital areas, with a broad region of elevated feature counts around Cz. θ-band HAC showed largely parallel selection frequency to rBP, whereas δ-band HAC exhibited increased selection frequency at C3/C4 and Oz. Within the β band, selection frequency demonstrated right-hemispheric predominance, with the highest concentration at O2, followed by elevated counts in frontal regions and other right-lateralized central and frontal locations.

In the higher frequency bands (β and γ), HCP did not reveal distinct spatially dominant areas. HFD demonstrated increased selection frequency in occipital, temporal, and frontal regions. This pattern partially overlapped with HMB but shared only left-frontal and right-temporal prominence with rBP. KFD, similar to HCP, did not exhibit prominent feature positions in the β or γ bands. PFD indicated weaker but still relevant information in the β band. Similar to HMB, occipital, frontal, and temporal feature counts were elevated; however, this specific band–feature combination showed overall lower counts compared to HMB or HFD. In the α band, PFD displayed a slight lateralized increase in selection frequency. RAB demonstrated moderate selection frequency, particularly in central and lateralized frontal regions, with a global increase across most electrode positions except occipital areas. RGT showed high similarity to the rBP pattern, with increased feature counts at distributed positions, especially occipital, temporal, and dorsofrontal regions. RTB exhibited moderate overall selection frequency, focusing on the frontal electrode row as well as selected occipital and left temporal sites. FAA was selected less frequently than RAB or any rBP feature. In contrast, FBA showed a strong global increase and ranked among the top four most frequently selected features overall.

**Figure 5:**
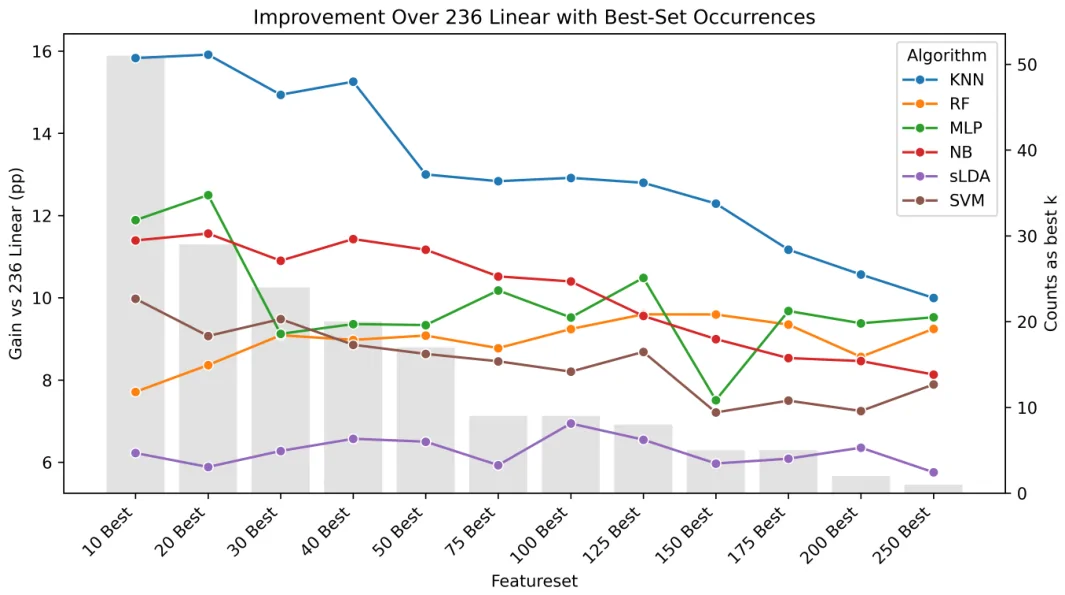
Feature-selection size and corresponding classification improvement across all training iterations of stress phase 1 versus stress phase 2. Grey bars show the number of training iterations in which each value of *k* was selected as the optimal K-Best feature set (right y-axis). Colored lines show, for each classifier, the average accuracy improvement in percentage points relative to the conventional feature set containing 236 features (left y-axis).

Figure 5 summarizes the distribution of the selected K-Best feature-set sizes across all participants and repeated random splits, together with the corresponding accuracy improvement relative to the conventional 236-feature reference set. The grey histogram shows how often each value of *k* was selected as the optimal feature-set size across the 150 evaluations. Thus, a higher count indicates that the corresponding value of *k* was selected more frequently during model optimization. Smaller feature sets were selected most often, with k=10 occurring 52 times and k=20 occurring 29 times, whereas larger values of *k* occurred considerably less frequently. The histogram therefore reflects the frequency with which a given feature-set size was selected as optimal and does not represent the proportion or contribution of a particular feature family. The relationship between feature-set size and accuracy improvement was classifier dependent. KNN showed the largest improvement at small feature-set sizes, reaching approximately 16 percentage points at k=10, and showed a general reduction in gain as *k* increased. RF exhibited a slight increase in accuracy gain between approximately 75 and 175 features, while the MLP showed a local decrease around k=150 and local maxima at smaller and intermediate feature-set sizes. sLDA varied only slightly across feature-set sizes, within a narrow range of approximately 5.8–6.8 percentage points. The remaining classifiers showed intermediate behavior. Overall, no consistent monotonic relationship between increasing *k* and classification gain was observed across all classifiers.

## 4. Discussion

The primary objective of this study was to compare the separation strength of established spectral and FD measures while evaluating their contribution to classification performance. The spatial feature-selection frequency maps were interpreted by relating them to the known behavior of the corresponding EEG features reported in the literature [24,25,28,30,32,61]. In addition, we examined the effect of statistical feature pre-selection on classification performance when combining conventional spectral features, Hjorth parameters, and FD measures.

### 4.1. Feature-Selection Frequency

In the rBP maps, we observed the most frequently selected features in the γ band with a global distribution, particularly across occipital, temporal, and frontal regions. In a stress-related task, Aspiotis et al. [64] reported overall activation in occipital β and γ bandpower and correlated these changes with stress indices. Furthermore, Meltzer et al. [65] associated similar activity patterns with highly cognitively demanding tasks, which aligns with our findings.

β rBP was frequently selected in frontal and occipital regions, supporting previous findings of altered β activity in frontal and parietal electrodes [9,16]. Additionally, our selection frequency maps are consistent with the original findings reported in [56]. Temporal selection frequency was also observed in [21]. While β rBP showed moderate asymmetry, this effect was more prominently reflected in FBA, where nearly all electrode pairs demonstrated high selection frequency. Although previous literature has often ranked FBA as less important compared to FAA [17], several studies highlight frontal β activity during cognitive processing and stress phases [9,12,20,56,66]. In our dataset, FBA demonstrated high selection frequency, consistent with the selected β rBP regions. Regarding the well-documented α suppression [9,12,16,20,21,64], we observed central α dominance in selection frequency. However, α rBP received less attention from classifiers compared to γ rBP and HAC. θ rBP exhibited generalized and low selection frequency, whereas δ-band rBP changes are more often associated with cognitive fatigue or high visual processing load [36]. As the δ-band did not show overall dominance, its selection frequency may relate to visual stimuli and increasing cognitive task difficulty. The highest overall selection frequency was observed in γ HAC. Its dominant selection frequency closely parallels γ rBP. Since γ rBP is strongly represented in HAC, this association is expected, whereas β rBP does not share this degree of overlap. HAC reflects activity patterns consistent with previous literature, including frontal, temporal, and occipital alterations [9,16,64,65]. The parallels between γ rBP and HAC are consistent with [23], suggesting that HAC may capture spectral changes in the β rBP in a more separable manner than rBP. α HAC again showed central dominance, potentially reflecting α suppression, as well as right-frontal and occipital contributions. θ HAC displayed moderate but distributed selection frequency similar to rBP, while δ HAC shared limited similarities with δ rBP, potentially reflecting central rhythms associated with fatigue. The high selection frequency of γ HAC is consistent with previously reported higher-frequency changes during cognitive load and stress [16,64,65]; however, its physiological origin cannot be determined uniquely from the present EEG recordings. However, this interpretation should be treated with caution. Although strict ICA-based artifact removal, filtering, and epoch rejection were applied, residual EMG contamination cannot be fully excluded. Although ICA-based artifact removal, visual and spectral inspection of muscle-related components, epoch rejection, and analyses excluding peripheral electrodes were applied, residual myogenic contributions in the γ band cannot be completely excluded. The reduction in classification performance after removing γ features therefore cannot be attributed uniquely to the loss of cortical activity, as it may also partly reflect removal of residual myogenic information.

HMB demonstrated moderate selection frequency primarily in the β band and less in γ. β HMB emphasized frontal, temporal, and occipital regions with slight asymmetry, resembling the β rBP pattern. γ HMB showed localized selection frequency in central and right temporal regions. As HMB reflects mean frequency characteristics, its spatial behavior aligns more closely with rBP than HAC. β HFD exhibited patterns similar to HMB, with frequent selection in frontal, symmetric temporal, and occipital regions. Temporal complexity thus appears to capture aspects comparable to β rBP, although we did not observe similar selection frequency in central regions. PFD showed comparatively low selection frequency. While β PFD displayed limited relevance in occipital, frontal, and temporal regions, overall selection frequency was lower than HMB or HFD. Given that PFD emphasizes rapid fluctuations and irregular transitions, dominant band power changes during stress may not induce sufficiently distinct transitions to increase separability. HCP and KFD did not exhibit substantial selection frequency. As KFD targets waveform tortuosity and HCP measures deviation from a sine wave, their conceptual overlap may explain their limited distinct contribution. While robustness to noise may be advantageous, both parameters appear to contribute less discriminative information for stress classification. Overall, FD measures did not exhibit strong selection frequency in δ, θ, or α bands. However, band-specific splitting revealed differential spatial distributions and feature contributions. Note that in the present study, FD measures were estimated after conventional band-pass filtering to investigate whether the complexity of specific oscillatory components provides complementary information for stress classification. We do not interpret these band-specific estimates as measures of global scale invariance or true fractality of the EEG signal. Rather, they quantify the irregularity and temporal complexity of oscillatory activity within a restricted frequency range, thereby allowing the relative contributions of different neural rhythms to be examined.

FBA emerged as a prominent relative feature despite the strong rBP presence. FAA did not demonstrate high selection frequency, although it has been frequently reported in cognitive processing [66,67,68]. RAB showed elevated selection frequency, particularly in central regions, reflecting combined α and β contributions. RTB demonstrated moderate selection frequency over frontal and isolated temporal and occipital regions.

The highest frequent features beyond rBP were observed in β and γ bands, particularly HAC, HMB, HFD, RGT, and FBA. These features were repeatedly selected in configurations that achieved stable and competitive classifier performance. Our findings partially align with [26]: selection frequency was weighted toward HAC and rBP. Given their similarity, HAC appears to carry substantial discriminative information. However, ANOVA-based pre-selection may bias ranking toward HAC, suggesting that alternative selection strategies should be considered in future work (e.g., mutual information based ranking). HAC and rBP may obtain high ranks in ANOVA-based feature selection because they capture signal variance and spectral distribution changes that differ systematically between experimental conditions [69]. Since ANOVA ranks features according to the ratio of between-class to within-class variance, features that show consistent condition-related differences tend to achieve higher F-scores and thus higher selection ranks. FD measures, particularly in the β and γ bands, occurred among the selected features in combined feature configurations that achieved higher performance than the conventional reference configuration. Visualization across features and bands further identified spatial regions relevant for stress classification and highlighted FBA as a potential marker during cognitively demanding stress conditions.

### 4.2. Classification Performance

The highest overall F1-score was achieved using RF with the split feature configuration. Band-specific feature extraction improved overall performance and reduced variability in classification performance across participants. Although KNN exhibited stronger variability, it achieved the highest average F1 when using all electrodes and band-specific features. RF outperformed SVM overall, demonstrating the smallest interquartile range, highest F1-score, and competitive standard deviation.

Our average performance ranged from approximately 74% to 85%, aligning with the current state of the art in stress detection [23,24,34,35,36] and exceeding recent reports [33]. Stable performance above 80% was achieved across classifiers, with peak performances reaching up to 98%. Given the balanced sex distribution in the dataset, consistent performance further supports the robustness of this individualized approach.

Excluding γ features resulted in a slight average accuracy reduction of approximately 7% and increased variability in performance across participants. However, overall performance remained stable, demonstrating robustness in a reduced feature configuration.

### 4.3. Effect of Feature Selection Size

When analyzing the number of selected features, we evaluated the average accuracy improvement relative to the conventional 236-feature reference set. Smaller feature subsets were selected most frequently during model optimization, with k=10 and k=20 representing the most common optimal configurations across participants and repeated random splits. This indicates that relatively compact feature sets were often sufficient to achieve high classification performance. Similar behavior has been reported in [35,38], where performance also decreased or saturated when increasingly large feature sets were used.

However, the relationship between feature-set size and classification gain was classifier dependent rather than uniformly decreasing. KNN showed the largest improvement for small feature sets, reaching approximately 16 percentage points at k=10, followed by a general reduction in gain as the number of features increased. In contrast, RF showed a slight improvement across intermediate feature-set sizes, whereas MLP exhibited local variations and sLDA remained comparatively stable over the investigated range. NB and SVM showed intermediate behavior without a pronounced monotonic trend. These differences likely reflect the varying sensitivity of the classifiers to feature dimensionality, redundancy, and model regularization.

This classifier-dependent behavior may reflect differences in how the algorithms handle increasing dimensionality, redundant or weakly informative features, and regularization. KNN relies directly on distances in the feature space and may therefore be particularly sensitive to the addition of less informative dimensions, which could explain the clearer reduction in gain with increasing *k*. In contrast, sLDA incorporates shrinkage regularization, which can stabilize estimation as the number of features increases and is consistent with its comparatively stable performance across feature-set sizes. The MLP also incorporates several regularization mechanisms, including dropout, batch normalization, AdamW with weight decay, and early stopping based on validation loss, which may reduce its sensitivity to increasing feature dimensionality. RF may likewise be comparatively tolerant of additional or redundant features because of its ensemble-based structure. These interpretations provide possible explanations for the observed classifier-specific patterns but were not directly tested in the present analysis.

Overall, the results suggest that increasing the number of selected features does not consistently improve classification performance and that the optimal feature-set size depends on the classifier. The frequent selection of small values of *k* nevertheless indicates that much of the discriminative information can be retained using relatively compact feature subsets. This is consistent with previous work showing that feature pre-selection can improve EEG-based classification by reducing redundant or weakly informative inputs [35,38]. This finding is also broadly consistent with previous studies showing that selected complexity-related and FD measures can provide useful information alongside conventional spectral features [34,36,37].

### 4.4. Key Findings and Implications

We carried out a fine-grained feature-selection frequency comparison and performance evaluation to obtain a broader understanding of the role of recently proposed FD measures. Compared with widely used EEG descriptors, KFD and PFD did not exhibit the same discriminative power as HAC, HMB, or HFD across the investigated frequency bands. These findings suggest that FD measures should not be considered a homogeneous feature class, as their ability to capture stress-related signal characteristics varies substantially across algorithms and frequency ranges. Furthermore, our analysis revealed a clear frequency dependence, with the most frequent FD measures predominantly originating from higher-frequency EEG bands. This observation is consistent with the expected behavior of complexity measures such as HFD and HMB, whose estimates are strongly influenced by local signal fluctuations.

In addition, we examined the spatial distribution of band-specific selection frequency across the scalp. This analysis revealed similarities and differences in the spatial and frequency-specific selection patterns of HAC, HMB, HFD, and rBP. Overall, the proposed classification framework achieved competitive classification performance, showing that FD measures can participate in effective combined feature representations. To assess potential artifact dependency, we further evaluated the framework under reduced feature conditions, including the removal of specific EEG bands or electrode groups. Even under these constraints, classification performance remained largely stable, indicating that the framework is not overly dependent on a single feature group.

The outcome of this work provides several insights: Firstly, the topographical information about selection frequency can improve future assessments of stress classifications with a reduced electrode layout. It also raises cautiousness about using electrodes at peripheral positions while retrieving artifact sensitive features. Secondly, our results highlight the continued selection frequency of physiologically interpretable spectral features. Combined feature-space configurations containing Hjorth parameters and FD measures achieved higher classification performance than the conventional reference configuration; however, the contributions of the individual feature families cannot be separated into unique and redundant components by the present analysis. Thirdly, the identification of a compact set of highly informative features may facilitate the development of neuro-adaptive systems that combine low computational requirements with robust performance. Finally, the presented framework may provide a useful basis for future investigations of related mental states such as fatigue, workload, and emotion regulation, thereby supporting the development of individualized EEG feature-selection strategies.

Our findings also raise several open questions that warrant further investigation. In particular, it remains unclear how the observed relationships between spectral features, Hjorth parameters and FD measures evolve under different cognitive conditions. While our analysis focused on a stress-inducing arithmetic paradigm, other cognitive states such as sustained attention, fatigue, or motor preparation may alter the relative contribution of spectral power and temporal complexity measures. For example, tasks with stronger oscillatory modulation might further emphasize band-power features, whereas conditions involving more irregular or transient neural dynamics could increase the relevance of FD measures. Understanding how these feature relationships change across paradigms could provide deeper insights into the neurophysiological mechanisms underlying cognitive state classification.

Another important question concerns the generalization of the proposed framework across participants. In the present work, the classifiers were trained and evaluated in an individualized setting, which typically yields higher classification performance due to subject-specific signal characteristics. The setup was chosen on purpose, as the long term goal to apply it in neuro-adaptive systems requires individualization rather than generalization. However, EEG signals exhibit substantial inter-individual variability in amplitude, spectral distribution, and spatial organization. Consequently, it remains unclear how robust the observed selection frequency patterns would remain under cross-participant or subject-independent training conditions. Future work should therefore evaluate the framework in cross-subject scenarios to determine whether the identified relationships between rBP, Hjorth parameters, and FD measures remain stable across individuals.

An additional aspect relates to the potential influence of EMG artifacts, particularly in the higher-frequency EEG bands. Although strict ICA-based artifact removal, filtering, and epoch rejection were applied, some residual EMG contributions cannot be entirely ruled out. This consideration is relevant because γ activity and peripheral electrode locations may be susceptible to muscular artifacts. At the same time, many EEG-based stress detection studies include γ power features as informative markers of cognitive load and stress [14,16,39]. The inclusion of the γ band was also motivated by its frequent use in previous EEG-based stress studies. Accordingly, the present analysis was intended not only to assess the contribution of γ-band information to classification performance, but also to examine whether the observed benefit of high-frequency features may depend partly on peripheral or potentially myogenic activity. The present findings therefore highlight the need for caution when interpreting γ-band activity as an EEG marker of stress. Nevertheless, further investigation may help clarify the relative contribution of neural γ activity and possible muscular influences. Future work could address this aspect by incorporating dedicated EMG recordings or by applying more advanced artifact separation techniques.

Beyond the specific influence of high-frequency and potentially myogenic activity, the permutation-based sensitivity analysis provides additional evidence for the relevance of the Hjorth and FD feature families within the combined feature representation. Shuffling either feature family reduced classification performance across all classifiers in the Stress Phase 1 versus Stress Phase 2 contrast, with a substantially larger reduction after permutation of the Hjorth parameters. This indicates that the trained models were sensitive to perturbation of both feature families, particularly the Hjorth parameters. The smaller but consistent reduction following permutation of the FD measures likewise indicates model reliance on these features within the combined representation. However, the permutation analysis does not establish that either feature family provides independent predictive information beyond conventional spectral features, nor does it fully separate unique from redundant information.

Beyond these aspects, the interactions between spectral features, Hjorth parameters and FD measures themselves merit further exploration. Our results suggest that HFD and Hjorth parameters capture information that partially overlaps with spectral power changes in the β and γ bands. Although the permutation analysis demonstrates classifier reliance on both feature families, it remains unclear to what extent this information is unique, complementary, or redundant with conventional spectral features. Investigating these relationships more systematically (e.g., through redundancy analysis or feature interaction studies) could help clarify the unique contribution of Hjorth parameters and FD measures to EEG-based classification tasks. At the same time, the feature-selection frequency maps assigned substantially lower selection frequencies to most FD measures than to physiologically interpretable markers such as Hjorth activity, frontal β asymmetry, and the γ/θ ratio. These findings show that most FD measures occurred less frequently among the highest-ranked ANOVA features than Hjorth activity, frontal β asymmetry, and the γ/θ ratio.

### 4.5. Limitations

Although our results represent a promising outlook, some limitations have to be taken into account. First, the experimental dataset consisted of recordings from 30 participants. While this sample size is comparable to many EEG-based experimental studies, larger cohorts would provide a broader basis for evaluating robustness across participants. Future investigations could therefore benefit from larger sample sizes and balanced sex distributions to enable more reliable sex-specific analyses.

Second, only a limited number of studies have systematically investigated selection frequency in EEG-based stress classification. As a result, the available literature provides relatively little evidence for such a direct comparisons across different feature families. Consequently, the interpretation of the relative selection frequency of spectral features, Hjorth parameters, and FD measures remains partly exploratory.

Third, additional research is needed to better understand the statistical relationships between commonly used spectral features such as rBP, Hjorth parameters such as HAC and FD measures. In particular, it remains unclear to what extent these feature groups provide complementary information or represent alternative representations of similar underlying signal characteristics. More systematic analyses of redundancy and interaction effects between these features may help clarify their respective contributions to classification performance.

The fourth aspect concerns the population under investigation. Additional insights may be gained by evaluating the proposed framework in populations with heightened stress sensitivity. Clinical studies including participants with specific phobias, anxiety disorders, or increased stress susceptibility could provide a clearer understanding of how the identified EEG patterns relate to stronger or more consistent stress responses.

Finally, the interpretation of the experimental conditions as stress-related is supported by the subjective measures obtained during the original experiment. BMIS scores showed a significant deterioration in overall mood following the experiment, while PASA stress scores increased significantly from the control condition to both stress phases. These findings support successful stress induction during the paradigm. Nevertheless, the experimental manipulation simultaneously altered task difficulty, time pressure, background noise, cognitive workload, and arousal. Consequently, the EEG differences identified in the present study should be interpreted as features associated with a stress-inducing cognitive task rather than as markers that are uniquely specific to stress.

Despite these limitations, the results of this study are promising. The proposed framework achieved competitive classification performance while providing detailed insights into the spatial and frequency-dependent relevance of spectral features, Hjorth parameters and FD measures. In particular, configurations incorporating FD measures and Hjorth parameters achieved higher classification performance than the conventional-feature reference configuration and helped reveal relationships between spectral power, Hjorth parameters, and fractal complexity measures.

Overall, our findings highlight the potential of combining spectral features, Hjorth parameters and FD measures for stress detection and provide a foundation for future work investigating feature interactions, individualized stress detection, and applications in more diverse cognitive and clinical settings.

## 5. Conclusions

This work presents a systematic comparison of the spatial selection frequency of band-specific FD measures and established EEG features used in stress classification. Within the investigated stress-inducing cognitive task, the findings improve our understanding of how different FD measures contribute across frequency bands and demonstrate that FD measures can be incorporated alongside physiologically interpretable spectral features in effective combined feature representations. The proposed analysis framework provides a basis for future research on interpretable EEG feature representations and individualized neuroadaptive applications.

## Figures and Tables

**Table 1:** Comparison of representative EEG-based stress-classification studies and the present work. Reported performance values correspond to the metrics and validation procedures used in the respective publications and are therefore not directly comparable across studies.

Study	EEG Data/Paradigm	Features	Classifier(s)	Reported Performance
Subhani et al. [20]	EEG recorded during the Montreal Imaging Stress Task; binary and multi-level stress classification	Absolute and relative spectral power, amplitude asymmetry, coherence, and related frequency-domain features	LR, SVM, NB	94.6% accuracy for two-level and 83.4% for multi-level stress classification
Arsalan et al. [15]	Four-channel Muse EEG from 28 participants; perceived-stress labels; two- and three-class classification	PSD, correlation, differential asymmetry, rational asymmetry, and power-spectrum features across frequency bands	SVM, NB, MLP	92.85% accuracy for binary and 64.28% for three-class classification
Saeed et al. [16]	Five-channel resting-state EEG; long-term stress labels based on perceived-stress scores and expert evaluation	Frequency-domain features and frontal/temporal α- and β-asymmetry measures	SVM and other ML classifiers	Up to 85.20% accuracy with expert-based labeling
Kamińska et al. [12]	EEG recorded during alternating relaxing and stressful VR conditions; Stroop task used as stressor	Frequency-band EEG information evaluated individually and jointly	CNN, MLP, SVM, conventional ML	Up to 96.42% accuracy when information from all EEG bands was used
Hamid et al. [34]	EEG recorded during the Stroop Colour Word Test with baseline and different stress conditions	FD measures compared with wavelet-based features	SVM	Mean F1-score of 83.03% for baseline–stress discrimination and 83.36% between stress conditions
Al Shorman et al. [49]	Frontal-lobe EEG recorded under stress and control conditions	FFT-derived spectral power-density features across EEG frequency bands	SVM, NB	Average subject-wise accuracy of 98.21%
Chatterjee et al. [51]	DASPS EEG dataset; 23 participants exposed to stress-inducing scenarios	Spatio-temporal transition representations derived from EEG features	Machine-learning classifiers	Maximum accuracy of 83.8% for both binary and four-class classification
Al-Shargie et al. [52]	EEG during rest, control-alert, Stroop-induced stress, and stress-mitigation conditions	Functional-connectivity features based on phase-locking value (PLV) represented as connectivity images	CNN	80.95% accuracy and 81.96% F1-score for four-state classification
Present study	28-channel EEG during a mental-arithmetic stress paradigm	rBP, BP ratios, frontal asymmetry, Hjorth parameters, and broadband and band-specific FD measures	sLDA, KNN, SVM, RF, NB, MLP	Best average F1-score of 84.67%

**Table 2:** Summary of all algorithms for feature extraction. The descriptive formulas describe the original definition, the specific implementation was done in python following those formulas.

Feature	Formula
HAC	$activity=\frac{1}{N}\sum_{i=1}^{N}{\left(x_{i}-\mu \right)}^{2}$
HMB	$mobility=\sqrt{\frac{var\left(\frac{dy_{\left(t\right)}}{dt}\right)}{var\left(y_{\left(t\right)}\right)}}$
HCP	$complexity=\frac{Mobility\left(\frac{dy_{\left(t\right)}}{dt}\right)}{Mobility\left(y_{\left(t\right)}\right)}$
KFD	$fdk=\frac{log(w)}{log\left(w\right)+log\left(\frac{d}{L}\right)}$
	*L* is sum of euclidean distances; *d* is diameter of subset F
HFD	$L_{m}\left(k\right)=\frac{1}{k}\left[\frac{N-1}{\left\lfloor \frac{N-m}{k}\right\rfloor k}\left(\sum_{i=1}^{\left\lfloor \frac{N-m}{k}\right\rfloor }\left|x\left(m+ik\right)-x\left(m+\left(i-1\right)k\right)\right|\right)\right]$
	$L\left(k\right)=\frac{1}{k}\sum_{m=1}^{k}L_{m}\left(k\right)$
	pick best fitting linear function through: $\left\{log\left(\frac{1}{k}\right),log\left(L(k)\right)\right\}$
PFD	$PFD=\frac{log_{10}\left(N\right)}{log\left(N\right)+log_{10}\left(\frac{N}{N+0.4N_{\delta }}\right)}$
	*N* is length of time series; $N_{\delta }$ is Number of sign changes
BP Ratio	$rab=\frac{S_{\alpha }}{S_{\beta }},rtb=\frac{S_{\theta }}{S_{\beta }},rgt=\frac{S_{\gamma }}{S_{\theta }}$
rBP: δ, θ, α, β, γ	$S_{xx(\omega )}=\lim_{T\to \infty }{\left|X_{T(\omega )}\right|}^{2}$
	$\mu_{FFT}=\frac{1}{N}\sum_{\omega =0.5}^{45}$
	$S_{rel,X}=\frac{S_{xx(\omega )}}{\mu_{FFT}}$
FAA, FBA	$faa=log_{10}\left(S_{left,\alpha }\right)-log_{10}\left(S_{right,\alpha }\right)$ $fba=log_{10}\left(S_{left,\beta }\right)-log_{10}\left(S_{right,\beta }\right)$

**Table 3:** Listing of each algorithm with the respective hyperparameter setting tuned per training iteration and electrode subset. The K-Best also is considered as optimized Hyperparameter, but not listed here as it tuned for all algorithms.

Algorithm	Hyperparameter
sLDA	number of estimators, solver, singular value tolerance
KNN	number of neighbors (3–10), weights, leaf size (10–40)
SVM	C (0.1–1000), gamma (1–1 × 10^−4^)
RF	number of estimators (100, 200, 300)
NB	Alpha (1, 0.9, 0.8)
MLP	batch size (8, 16, 32)

**Table 4:** F1 scores (average: avg, standard deviation: std) for all classifiers and feature configurations: Conditions used were Stress Phase 1 and Stress Phase 2. The best performances overall are highlighted.

Feature Config	F1 Avg	F1 Std	Feature Config	F1 Avg	F1 Std
NB			RF		
No ring	76.22	11.27	No ring	79.71	12.28
Band-specific	79.36	11.32	**Band-specific**	**84.67**	**10.53**
No γ	74.47	11.99	No γ	79.33	11.06
KNN			SVM		
No ring	75.48	14.51	No ring	77.28	13.13
Band-specific	80.58	13.20	**Band-specific**	**83.94**	**10.23**
No γ	74.39	12.45	No γ	75.33	14.61
MLP			sLDA		
No ring	78.37	12.50	No ring	77.49	14.36
Band-specific	82.48	10.55	Band-specific	82.63	11.31
No γ	75.29	13.02	No γ	76.02	13.12

## Data Availability

The analysis code supporting the findings of this study and the dataset are available from the corresponding author upon reasonable request.

## Abbreviations

The following abbreviations are used in this manuscript:

ANOVA	Analysis of Variance
BCE	Binary Cross-Entropy
BMIS	Brief Mood Introspection Scale
BP	Band Power
ECG	Electrocardiogram
EEG	Electroencephalogram
EMG	Electromyography
FAA	Frontal Alpha Asymmetry
FBA	Frontal Beta Asymmetry
FD	Fractal Dimension
FDR	False Discovery Rate
FFT	Fast Fourier Transform
FN	False Negative
FP	False Positive
HAC	Hjorth Activity
HCP	Hjorth Complexity
HFD	Higuchi Fractal Dimension
HMB	Hjorth Mobility
ICA	Independent Component Analysis
KFD	Katz Fractal Dimension
KNN	k-Nearest Neighbors
LSL	Lab Streaming Layer
MLP	Multi-Layer Perceptron
NB	Naive Bayes
PASA	Primary Appraisal–Secondary Appraisal
PFD	Petrosian Fractal Dimension
PSD	Power Spectral Density
RAB	Alpha/Beta Band-Power Ratio
rBP	Relative Band Power
RF	Random Forest
RGT	Gamma/Theta Band-Power Ratio
RTB	Theta/Beta Band-Power Ratio
sLDA	Shrinkage Linear Discriminant Analysis
SVM	Support Vector Machine
TN	True Negative
TP	True Positive
